# Mechanisms of Communicating Health Information Through Facebook: Implications for Consumer Health Information Technology Design

**DOI:** 10.2196/jmir.5949

**Published:** 2016-08-11

**Authors:** Hannah K Menefee, Morgan J Thompson, Thomas M Guterbock, Ishan C Williams, Rupa S Valdez

**Affiliations:** ^1^ Department of Population Health Sciences Virginia Tech Blacksburg, VA United States; ^2^ Department of Psychology The College of William and Mary Williamsburg, VA United States; ^3^ Department of Public Health Sciences; Center for Survey Research; Department of Sociology University of Virginia Charlottesville, VA United States; ^4^ School of Nursing University of Virginia Charlottesville, VA United States; ^5^ Department of Public Health Sciences University of Virginia Charlottesville, VA United States

**Keywords:** consumer health information, medical informatics, Facebook, social networks, health communication

## Abstract

**Background:**

Consumer health information technology (IT) solutions are designed to support patient health management and have the ability to facilitate patients’ health information communication with their social networks. However, there is a need for consumer health IT solutions to align with patients’ health management preferences for increased adoption of the technology. It may be possible to gain an understanding of patients’ needs for consumer health IT supporting their health information communication with social networks by explicating how they have adopted and adapted social networking sites, such as Facebook, for this purpose.

**Objective:**

Our aim was to characterize patients’ use of all communication mechanisms within Facebook for health information communication to provide insight into how consumer health IT solutions may be better designed to meet patients’ communication needs and preferences.

**Methods:**

This study analyzed data about Facebook communication mechanisms use from a larger, three-phase, sequential, mixed-methods study. We report here on the results of the study’s first phase: qualitative interviews (N=25). Participants were over 18, used Facebook, were residents or citizens of the United States, spoke English, and had a diagnosis consistent with type 2 diabetes. Participants were recruited through Facebook groups and pages. Participant interviews were conducted via Skype or telephone between July and September 2014. Data analysis was grounded in qualitative content analysis and the initial coding framework was informed by the findings of a previous study.

**Results:**

Participants’ rationales for the use or disuse of a particular Facebook mechanism to communicate health information reflected six broad themes: (1) characteristics and circumstances of the person, (2) characteristics and circumstances of the relationship, (3) structure and composition of the social network, (4) content of the information, (5) communication purpose, and (6) attributes of the technology.

**Conclusions:**

The results of this study showed that participants consider multiple factors when choosing a Facebook mechanism for health information communication. Factors included what information they intended to share, what they were trying to accomplish, attributes of technology, and attributes and communication practices of their social networks. There is a need for consumer health IT that allows for a range of choices to suit the intersectionality of participants’ rationales. Technology that better meets patients’ needs may lead to better self-management of health conditions, and therefore, improve overall health outcomes.

## Introduction

Recent government initiatives encouraging the development and implementation of health information technology (IT) have expanded the definition of health care delivery locations to include home and community environments [1]. With easier and increasing access to health IT solutions, patients’ interactions with their health information and health systems can now occur outside of the clinical encounter [1]. Patients’ engagement in their health care, or patients having the knowledge, skills, confidence, and tools to manage their health, has proven beneficial for improving long-term health outcomes [2,3]. Consumer health IT may be one means of supporting patient engagement [4,5]. Although there is no consensus definition of consumer health IT [6], the term generally refers to electronic technology designed specifically to support laypeople with health and health care management. These technologies can position patients to actively participate in their care by facilitating self-management of their conditions and improve coordination and communication among their care team [1,3].

It is imperative that consumer health IT is designed to align with patients’ health management needs [7-11]. One challenge that patients with chronic conditions face is effectively engaging others in their health management [12-15]. Successful health management often involves communicating health information with a larger social network (eg, friends, family members, others with similar diagnoses) [12-14,16]. Several consumer health IT solutions have the ability to support patients’ health information communication with members of their social network. Examples of these technologies include CaringBridge, Microsoft HealthVault, MyChart, and online health communities such as PatientsLikeMe, QuitNet, and TuDiabetes. However, these existing solutions are currently limited in relation to what, to whom, and how health information is communicated [14].

Designing consumer health IT that effectively supports health information communication between patients and their social network members requires gaining a deep understanding of existing communication practices. Such an effort may generate both descriptive (ie, narrative explication of communication approaches) and prescriptive (ie, specific design actions) design guidance [14]. Several studies have sought to understand the ways that patients communicate health information face to face, over the phone, through video chat, and using other common non‒social media based communication mechanisms for the purpose of drawing design guidance for consumer health IT [12-14,16,17]. Others have sought to draw such design guidance from patients’ communication practices within online health communities [18-21]. However, previous studies have only partially drawn consumer health IT design guidance from the ways that patients have adopted and adapted readily available information technology for health management, which are not specifically designed for this purpose (ie, from technologies that are not consumer health IT). These technologies include social networking sites (SNS), which have been adopted and adapted by patients for health management [22-26], including communicating health information with social network members [27-31].

In this study, we use Facebook as a case study from which to draw design guidance for consumer health IT that supports health information communication between patients and their social networks. Facebook was selected as an object of study because it is a general information technology that takes the form of an SNS rather than consumer health IT. Furthermore, its popularity, with 70% of site users engaged daily [32], and usage as a site for health communication [27,33] contributed to our choice. Facebook was also chosen as a case for this study because of the diverse communication mechanisms offered to users, which span those that may be used in ways that are public versus private, synchronous versus asynchronous, and active versus passive. This SNS enables communication with social networks defined by personal relationship (eg, friends) and shared health condition (eg, other group members). A list and brief discussion of these mechanisms is found in Multimedia Appendix 1. By understanding why patients choose one Facebook mechanism over another for health information communication, designers (ie, individuals creating consumer health IT solutions) can gain insight into what communication mechanisms should be replicated, abandoned, or modified for consumer health IT supporting health information communication between patients and their social networks. Such insight can then be synthesized with other assessments of participant needs and incorporated within a participatory design process [34] to create an acceptable consumer health IT solution for a particular population.

Previous research on health information communication on Facebook has primarily sought to understand how patients use the group mechanism. Patients’ interactions on health-related Facebook groups are structured around informational and emotional support and community building [23,35-39]. Medical and lifestyle information, including users’ personal experiences, opinions, and advice are frequently communicated and highly valued by users of health-related Facebook groups [35,36,38,39]. In addition to informational support, emotional support in the form of encouragement and affirmation to help motivate other users is frequently found in posts to health-related Facebook groups [36,38-40]. Informational and emotional support cultivates health-related Facebook groups as spaces for companionship and community [36,38]. Some health-related Facebook groups also develop group identity by rejecting common stereotypes to reframe their condition in a positive light [35]. Moreover, health-related Facebook groups often become spaces for the marketing of health services or products [23,41], enabling expression of experiences with health care centers, providers, and insurance companies [39], raising general awareness, and supporting fundraising efforts [22,37,41]. Users of health-related Facebook groups also include more than just patients. Family members, advertisers, and researchers participate and often communicate health information within these groups as well [23,39].

While these previous studies make meaningful contributions to our understanding of how patients engage with Facebook groups for health information communication, they lack insight into how and why patients engage with other Facebook mechanisms to communicate information about their health. Expanding analysis efforts to encompass other Facebook mechanisms enables understanding of how patients communicate not only with others with similar diagnoses (ie, within Facebook groups), but how they communicate health information with their larger social networks (ie, with Facebook friends and friends of friends). Accordingly, the purpose of this study was to characterize patients’ rationales for using diverse communication mechanisms within Facebook for health information communication, each of which presents the Facebook user with a choice regarding who is the target audience, whether the communication is synchronous or asynchronous, and whether the communication is relatively active or passive. From this analysis, we hope to provide insight into how consumer health IT may be better designed to meet patients’ health information communication needs and preferences.

## Methods

### Study Details

This study analyzed data from a larger, three-phase, sequential, mixed-methods study. The larger study’s first phase used in-depth participant interviews (N=25) to gain a broad understanding of patients’ health information communication practices on Facebook. These interviews are the focus of this paper.

### Sample

Eligible study participants self-reported in a pre-study survey that they were over 18, used Facebook, were residents or citizens of the United States, spoke English, and had a diagnosis consistent with type 2 diabetes. Type 2 diabetes was the focus of this study because it is a chronic disease that is highly prevalent, requires intense personal engagement, and disproportionately affects underserved populations [14]. The University of Virginia’s (UVa) Social and Behavioral Sciences Institutional Review Board approved this study, and all participants provided informed consent.

### Recruitment

The recruitment process has previously been described in detail [42]. In short, we began by identifying diabetes-related Facebook groups and pages as well as those specific to racial and ethnic minorities. The latter were targeted to promote sample diversity. Consenting moderators posted a link to, and information about, our study’s Facebook group. Each member of our Facebook group was asked to complete an online pre-study survey, deployed via Qualtrics [43] by UVa’s Center for Survey Research. The pre-study survey focused on demographic characteristics, including race, ethnicity, gender, socioeconomic status, geographic location, and frequency of Facebook use. Maximum variance sampling [44] based on these demographic characteristics was used to target individuals for interview participation. Participants were provided US$25 in compensation.

### Data Collection

Semistructured interviews were conducted via Skype or telephone between July and September 2014 and ranged from approximately 21 minutes to 68 minutes in length (average=43 minutes). Participants were asked about their general use of Facebook (eg, “How large is your social network on Facebook?”), how they used specific Facebook mechanisms (eg, “Do you chat or send private messages? Why or why not?”), and their Facebook-related privacy concerns and settings (eg, “What concerns, if any, do you have regarding your privacy on Facebook?”). Participants were next asked systematically about components of their health information communication work system, including to whom and why health information is communicated (ie, social subsystem), what and how health information is communicated (ie, technical subsystem), and how factors such as their economic, political, cultural, and health statuses impacted their health information communication practices (ie, external environment) [45,46]. All interviews were audio recorded, professionally transcribed, and reviewed by the research team prior to analysis.

### Data Analysis

Data analysis was grounded in qualitative content analysis [47] and facilitated by the qualitative analysis software program QSR NVivo 10 [48]. The unit of analysis was the full response provided by the participant for each question. Simultaneous coding was used when units of analysis contained multiple meanings [49]. The initial coding framework was informed by the findings of a previous study [50] and was revised based on participant responses in this study. The original framework included the six broad themes (ie, person, relationship, third party, message, goals, context) as well as categories under each theme. Data from this study were deductively coded into these existing themes and categories. When a unit of analysis did not fit into a preexisting theme and/or category, a new theme and/or category was created. Two investigators independently coded the interviews (HKM & MJT), and discrepancies were referred to the corresponding author (RSV) for resolution. The three authors discussed any coding disagreements. Outcomes of the discussions were used to revise the coding framework.

## Results

### Sample Characteristics

A total of 25 individuals participated in the interviews. Most were female and between the ages of 30 and 64. All major racial and ethnicity categories were represented. The majority of participants were white and non-Hispanic. All participants had received at least a high school diploma or equivalent and most participants worked full time (35 hours/week or more). Individuals from all marital statuses, household incomes, and geographic regions were represented. The majority of participants were married, had a yearly household income between US$75,000 and $149,000 and were from suburban areas. Individuals represented all brackets of self-rated health status and years with type 2 diabetes. Over half of the participants rated their health as good and had been diagnosed with diabetes for 3-19 years. Most participants used Facebook more than once a day. Sample characteristics are shown in Table 1.

### Participants’ Rationales for Health Information Communication Mechanism Use

Participants’ rationales for the use of a particular mechanism to communicate health information reflected six broad themes. Each theme represents the types of rationales provided for communicating or not communicating health information through a specific Facebook mechanism: (1) characteristics and circumstances of the person, (2) characteristics and circumstances of the relationship, (3) structure and composition of the social network, (4) content of the information, (5) communication purpose, and (6) attributes of the technology. Participants’ responses to open-ended questions are presented verbatim to provide evidence for analytical categories. The quotes selected are representative rather than exhaustive of the analytic category. It is important to note that the multidimensionality of participants’ rationales means that an illustrative quote may contain more than one meaning (and that, as stated above, quotes were often coded under multiple rationales).

#### Theme 1: Characteristics and Circumstances of the Person

Participants’ choice of Facebook communication mechanism reflected their own and their social network members’ characteristics and/or circumstances.

##### Individuals’ Time Available to Talk About Health

Participants reflected on the time they had available to use specific Facebook mechanisms for health information communication. Some participants noted that posting health information on one’s timeline would result in the post being available to view by most of their social network and that the publicity of such messages would result in undesired feedback and consume their time for response:

"I would post, ‘Hey, I have some kind of issue with my kidneys.’ All that’s gonna do is open up, ‘What kind of issue? Oh my gosh, are you okay? What are they gonna do?’ I have no room to answer a million questions."

173, F, Black or African American, 30-49

##### Individuals’ Degree of Health-Related Knowledge and Experience

Participants discussed the role of their and their social network members’ health-related knowledge and experience. They stressed the importance of not posting health information about which they had limited knowledge to their own or friends’ timelines on Facebook:

"[T]here’s a lot of stuff that comes in on a feed on your Facebook...I am not going to share something that I don’t know anything about."

69, F, Hispanic or Latino, Other race, 50-64

Participants also considered the health-related knowledge and similarities in experiences of individuals that could be reached through a specific Facebook mechanism:

"If I have an issue going on and maybe I want to know if someone else has experienced the same issues, I want to know if they tried out a certain product before I buy it. I may post that in the discussion board for that diabetes group."

90, F, Declined to answer race, 30-49

Moreover, participants stressed the ability to reach individuals with diverse knowledge and experience using the group mechanism:

"Groups has a wide variety of people, and they’ve got lots of information, different attitudes about it, different answers. I can get a wide variety from those people."

120, F, White, 50-64

##### Individuals’ Temperament and Mood

Participants noted that their overall dispositions played major roles in determining how they communicated health information on Facebook. Shyness, depression, and feeling tired all deterred participants from using public mechanisms of communication such as their own timeline or groups. Participants also recognized that specific moods altered their approaches to using these mechanisms for health information communication:

"Well, in terms of images of myself, personally, I’m a little insecure about my weight and that kind of stuff, so unless I’m really in a good mood or something...when I was relaxed or whatever, I wasn’t too shy about posting those kinda pictures—me sitting on the island or something like that. That’s the primary reason. Appearance."

11, M, Black or African American, 30-49

##### Individuals’ Patterns of Technology Use

Social network members’ patterns of communication mechanism use also influenced participants. Specifically, others’ degree of engagement with specific mechanisms impacted participants’ frequency and likelihood of use. Participants engaged with a communication mechanism when they knew that the social network member that they were trying to reach would be available through that mechanism:

"[Messenger is] a fast form of communication with some people like my Weight Watchers leader. She’s on messenger all the time."

185, F, White, 65+

Participants’ decision of whether or not to engage with communication mechanisms involving multiple individuals similarly depended in part on the level of group activity. Thus, one participant noted that even though there are many people in his Facebook groups, there are not many that are active members, and expressed:

"...if people were more active, or more communicative, I would be the same way."

11, M, Black or African American, 30-49

##### Individuals’ States of Well-Being

Participants’ states of well-being determined how they communicated health information through Facebook. Certain Facebook mechanisms deterred participants with physical limitations because of the normative length of the message exchanged via that mechanism:

"I can’t see well, and a lot of times my fingers hurt bad, and I don’t like to private message because of that."

120, F, White, 50-64

However, participants also expressed reaching out to others in their social network broadly through groups and posting on timelines when facing poor health:

"Primarily I’ve been well, since I signed onto Facebook. There’s always a chance that yeah, if I was feeling under the weather I would put a post on there, just so everyone would know."

130, M, White, 65+

#### Theme 2: Characteristics and Circumstances of the Relationship

Participants’ choice of Facebook communication mechanism reflected characteristics and circumstances of the relationships between them and their social network members.

##### Definition of Relationship

The roles that social network members played in participants’ everyday lives (ie, specific social or biological ties) influenced the choice of communication mechanism on Facebook. For example, participants posted on their own and friends’ timelines simply because of the nature of the relationship:

"[T]he choice of my friends are all people that have done the same thing I did, are working hard, making an income, taking care of themselves. The fact that they’re my friends is why I’m posting to them, and we have a lot in common."

130, M, White, 65+

##### Established Patterns of Relationship

The established communication patterns between participants and their social network members influenced the choice of Facebook mechanism for health information communication. Participants noted that they used certain Facebook mechanisms habitually to communicate to specific social network members and that their communication of health information to the same individuals followed this pre-existing routine:

"...once they know that there’s something, that you’ve got this problem, then talking back and forth on Facebook [messages]—because, as I said, that’s the way we do a lot of our conversing."

114, F, Black or African American, 50-64

Likewise, established communication patterns hindered the communication of health information via the same mechanisms:

"Pretty much the people that I message are not ones that I share the diabetes information with."

139, F, White, 30-49

#### Theme 3: Structure and Composition of the Social Network

Participants’ choice of Facebook communication mechanism reflected the structure and composition of participants’ and their social network members’ social networks.

##### Mutual Friends Within Social Networks

The degree of interconnectedness between participants and members of their social networks influenced the use of some Facebook mechanisms for health information communication. Often participants were concerned that the information posted on Facebook timelines would be brought into offline conversations with mutual friends:

"I would be very careful about what I’d say about someone else because so many of my friends are interconnected."

55, F, White, 65+

##### Friends of Friends Within Social Networks

Participants acknowledged that the ability of certain Facebook mechanisms to reach friends of friends was useful when wanting to provide support to other type 2 diabetes patients. A few mentioned sharing pages related to diabetes on their timelines for the benefit of people beyond their immediate Facebook social network:

"I share some pages for diabetes, cuz I do know some people that have it or maybe their spouses. If I see anything that I like, I like somebody else to see it. Cuz not everybody gets the same feed. I might share a page."

69, F, Hispanic or Latino, Other race, 50-64

##### Social Network Insufficiency

Participants stressed that certain Facebook mechanisms gave them access to information and support not available to them through existing social network members. This was particularly true of the group mechanism:

"I think the reason why I’m in those groups, though, is because I was kind of pissed off that there was really no help that was worth anything from my doctor. I mean she gave me some general stuff, but nothing that was of any substance. That’s kinda why I participate in the groups."

92, M, White, 30-49

#### Theme 4: Content of the Information

Participants’ choice of Facebook communication mechanism reflected the substance of the health information.

##### Gravity of Information

Participants considered the seriousness of their messages. For instance, one participant noted that she did not consider one piece of information to be major or serious and therefore felt the information was appropriate to communicate using Facebook messages:

"[Y]ou know, at that time, it didn’t make sense for me to sit and try to call 10 different people, I could do it in like two or three minutes on Facebook. It wasn’t something that was major, serious, or deathly, it was something that [my mother] had been through a number of times before."

114, F, Black or African American, 50-64

##### Personal Nature of Information

The degree to which participants considered the health information to be private influenced their use of Facebook mechanisms. For example, one participant felt that general health information unrelated to her diabetes was appropriate for her Facebook timeline, but that private health information should not be discussed using this mechanism:

"I wouldn’t talk about, like I said, anything that was, that I consider very, very, very private—but general stuff, I mean, if you look and you see my leg is broke, I don’t see why I can’t talk about a broken leg on Facebook."

114, F, Black or African American, 50-64

##### Sensitivity of Information

The emotional weight of the health information also influenced the use of Facebook mechanism. Participants acknowledged that some health information would invoke strong reactions from their social network members. The intensity of these messages determined which Facebook communication mechanism, if any, was used:

"[S]ay I had a diagnosis of cancer or anything, a diagnosis that I knew would really be shocking to someone, I would tell them face to face. I wouldn’t send my sister or brother a Facebook message and say, “Oh, by the way, I've got cancer.” I wouldn’t do that. I would personally call them."

114, F, Black or African American, 50-64

##### Specialized Nature of Information

A majority of participants described the use of Facebook groups when discussing a particular diabetes topic, expressing that this mechanism was more appropriate than discussing this type of detailed information on their timelines. Furthermore, participants explained the relationship between level of specialization and communication via Facebook groups:

"In the groups, it’s specifically for something special. I might be a member of the Rheumatoid Arthritis page, and we’ll be talking about methotrexate or something. If I posted something about methotrexate on my newsfeed, 80 percent of the people are not going to have a clue what I’m talking about…I can be more specific on the group pages."

120, F, White, 50-64

##### Relevance of Information

The Facebook mechanism used was also determined by the perceived novelty of the health information. When such information was perceived as relevant to many individuals, participants used the sharing mechanism or posted to their timelines:

"I may see something in a diabetes group on Facebook, go click on it, “Oh, that’s really interesting. I’m gonna share it,” and go back to Facebook and share it and put it on my page. That’s typically how I do it. On occasion, I’ll see something through another source, read it, “Oh that’s interesting,” and click on it and share it on Facebook that way."

139, F, White, 30-49

Participants also considered how relevant the health information was to what was typically communicated through the Facebook mechanism:

"Because it’s relevant to the group that I’m part of. Mostly because it’s relevant or it’s relevant to a conversation that’s occurring in the group or within an individual conversation."

33, F, White, 50-64

Even if participants believed in the relevance of the information to a specific individual, they sometimes used mechanisms that had a wider reach instead of imposing the information on a specific person:

"They’re just things [posted to own timeline] that I’m interested in and may be able to help other people. I don’t really—putting it on their timeline is kind of like ‘pushy’."

33, F, White, 50-64

##### Scarcity of Information

The existence and availability of health information to others in their Facebook network influenced participants’ use of mechanisms for communicating health information. Participants used mechanisms targeted to a wider social network when they believed the information they were sharing was not well known to this audience. For example, one participant focused on the relative lack of information regarding type 2 diabetes:

"I would say just because I feel like there is not enough people talking about type 2 diabetes, it shapes my posts in the sense that I post a lot about diabetes health. It has made me—my health status has made me an advocate for informing other people about type 2 diabetes."

173, F, Black or African American, 30-49

#### Theme 5: Communication Purpose

Participants’ choice of Facebook communication mechanism reflected the goals they sought to achieve in relation to their health information.

##### Ensure Personal Communication

Participants sought to use the Facebook mechanism they felt would ensure a more personal connection when communicating health information. For the majority of participants, preserving this connection was achieved by using the private messaging mechanism:

"It’s a private thing, and it’s personal; so people are more likely to respond to personal messages."

173, F, Black or African American, 30-49

##### Reduce Burden

Participants’ rationales for using specific Facebook mechanisms to communicate health information included reflection on the degree of convenience versus hardship on themselves and others. The ability to use certain Facebook mechanisms like messages to address a specific group was mentioned by participants as more convenient than other mechanisms requiring one-on-one interaction:

"Sometimes it’s easier to get a blanket statement out there, especially to the friends."

146, M, White, 30-49

Similarly, mechanisms that did not require sifting through previous communications were also seen as more convenient:

"If I had previously spoken to someone about a problem that I’ve had, or a problem that they have had. Then, that post is now I don’t know where, and I feel that I have an additional bit of information that they could use, then I might tag them on that. I write it on my timeline and just say, “So-and-so, I just thought I would share this with you."

3, F, Asian, 50-64

Private messages were also mentioned as a means of reducing discomfort that could come from confronting someone in a public forum such as a Facebook timeline:

"If I see somebody giving [misleading health information] or leading a person, I feel, astray, then I will send them a private message versus putting somebody on the spot right there, calling someone out."

114, F, Black or African American, 50-64

##### Connect With Others

The ability of the Facebook mechanism to allow for a message to reach the intended audience was a motivating factor for use by some participants. When considering the ability to obtain responses quickly, one participant explained:

"The groups are between 500 to 3,000 people…there’s going to be more people online at any given time to give you instant feedback."

33, F, White, 50-64

The participant also stated that having more group members helped him find:

"...somebody to sympathize with or to share information with or to get information from."

33, F, White, 50-64

Another participant posted on Facebook immediately after obtaining the result of an exam that indicated potential need for retinal surgery, so that his doctor would receive the message:

"I did that primarily because my eye doctor reads that, so that as soon as he sees it he’s gonna call me and we’re gonna talk about it on the phone."

130, M, White, 65+

Moreover, some participants noted that communicating health information on friends’ timelines would be considered a last resort if they could not reach members of their social network by other means:

"Maybe if I was dying then I would post something on my best friend’s timeline to get her attention if I could not reach her by telephone, or couldn’t reach her family or mom or anybody, then maybe I would do that. It would be a last resort."

90, F, Declined to answer race, 30-49

##### Preserve Personal Security

Participants reflected on the goal of maintaining online security when deciding to use one Facebook mechanism over another. In particular, some participants favored use of mechanisms that restricted communication to one or a few individuals in order to maintain security of their information:

"If I don’t want—if I’m on one of my group pages, and I don’t want all those strangers to know what’s going on, I would use the private messaging to go back and forth with someone.”

120, F, White, 50-64

Participants’ social network members also eased their online security concerns about communicating through more public forums, such as Facebook groups:

"It would depend. This particular instance, your group was referred to me by someone I knew very well. I felt comfortable doing that. I don’t necessarily trust other groups. I don’t know what their guidelines for their privacy and all that are."

120, F, White, 50-64

##### Manage Content Received

Participants used Facebook mechanisms that would ensure they obtained the content desired. Groups in particular were sources of desired information:

"On the groups I’ll post something because it’s a diabetes type 2 group and I’m sure that somebody is going through the same thing or been through it, and they’re going to tell me something that may help me out."

90, F, Declined to answer race, 30-49

Obtaining the health information or feedback desired was also achieved using Facebook mechanisms with the ability to target participants’ messages to certain social network members:

"Again, it’s situational. I don’t think everybody needs to know everything, so I target my message to who I think is interested or who I’ll get sympathy from. I can’t put something out there and have everybody go, “Poor baby, suck it up!” I want people to go, “Aww, it’s okay. It’ll be okay."

33, F, White, 50-64

Similarly, participants sought to avoid unwanted input and information by refraining from using certain Facebook mechanisms. One participant knew that if he posted his poor A1C level to his timeline he would:

"...get scolded by one friend who’s an eye doctor."

226, M, White, 50-64

##### Provide Support

Participants noted that some Facebook mechanisms facilitated their ability to provide instrumental and emotional support. One participant felt that by using the “share” mechanism to post diabetes-related information:

"someone else can read it, and can learn from it, and not feel that they’re in the daily management of diabetes by themselves."

173, F, Black or African American, 30-49

Participants also stressed commenting on posts as a form of providing emotional support.

##### Provide Response

Wanting or not wanting to address others’ inquiries, interests, and/or requests influenced participants’ choice of Facebook mechanism. In many cases, participants were comfortable responding to social network members’ questions using the mechanism by which the question was asked:

"If someone asks me about it, if someone from one of the groups or something or the pages asks me about it, I would probably communicate through that, in that way."

120, F, White, 50-64

However, other participants expressed situations in which other considerations outweighed their desire to respond to inquiries from their social network:

"Right now, I have an issue with my kidneys…I’m not posting that. It’s probably related to diabetes, but I don’t know. If it’s something that opens myself up to a bunch of questions, I don’t post it."

173, F, Black or African American, 30-49

#### Theme 6: Attributes of the Technology

Participants’ choice of Facebook communication mechanism reflected characteristics of specific Facebook mechanisms.

##### Mechanism Reliability

Participants considered the reliability of social network members receiving health information through a specific Facebook mechanism. For example, due to Facebook’s structure, depending on the time of day a user is logged into the site and/or the popularity of a particular post, certain posts may or may not appear in a user’s newsfeed. Therefore, participants depended on other means of sending a message:

"I know with the newsfeed thing, you have most recent and whatever the other one is, most popular, or something. I know that sometimes I could post something on my page, but it doesn’t show up in everyone’s newsfeed. Depending on when they look at Facebook, like it could be at the bottom, and they don’t see it. I will send private message just as confirmation if I wanna make sure that person got the information."

173, F, Black or African American, 30-49

##### Mechanism Integration With External Sites

Facebook is integrated with many external websites through the “share” mechanism. A few participants acknowledged the convenience of using the “share” Facebook mechanism on external websites to post useful health information to their timeline:

"They’re convenient to share. Usually it’s from a website with a share button."

172, M, Hispanic or Latino, American Indian/Alaskan Native, 30-49

##### Mechanism Reach

The structures of the private message and chat mechanisms on Facebook have the ability to easily facilitate communication with groups of people. Several participants acknowledged that these mechanisms were beneficial when they wanted to send out information to a large number of specific people in a short time:

"If something was—I needed to get out to a lot of people in a short period of time and sitting down making 20, 30, 40 phone calls was not something that I could do, then I could use Facebook and those friends and send a private message, because you can send a private message to groups of people, I’m able to do it that way."

114, F, Black or African American, 50-64

**Table 1:** Demographic characteristics of participants (N=25).

Sample characteristics		Valid % (n)
**Gender**		
	Male	44 (11)
	Female	56 (14)
**Age**		
	18-29	0 (0)
	30-49	44 (11)
	50-64	40 (10)
	65+	16 (4)
**Race**		
	White	64 (16)
	Black or African American	16 (4)
	American Indian/Alaskan Native	4 (1)
	Asian	4 (1)
	Native Hawaiian/Pacific Islander	0 (0)
	Other	4 (1)
	Declined to answer	8 (2)
**Ethnicity**		
	Hispanic or Latino	12 (3)
	Not Hispanic or Latino	88 (22)
**Education**		
	Less than high school	0 (0)
	High school diploma or equivalent	16 (4)
	Some college, but no degree	16 (4)
	Associate’s degree	8 (2)
	Bachelor’s degree	20 (5)
	Some graduate work	12 (3)
	Master’s degree	16 (4)
	Doctoral degree	4 (1)
	Professional degree	8 (2)
**Employment status**		
	Working full time (≥35 hours/week)	48 (12)
	Working part time	20 (5)
	Looking for work	4 (1)
	Homemaker	0 (0)
	Retired	20 (5)
	Student	0 (0)
	Other (eg, disabled)	8 (2)
**Marital status**		
	Married	68 (17)
	Widowed	4 (1)
	Divorced	16 (4)
	Separated	4 (1)
	Never married	8 (2)
**Yearly household income, USD**		
	<$30,000	16 (4)
	$30,000-$49,999	16 (4)
	$50,000-$74,999	12 (3)
	$75,000-$149,999	36 (9)
	≥$150,000	8 (2)
	Decline to answer	12 (3)
**Geographic region**		
	Urban	24 (6)
	Suburban	40 (10)
	Rural	32 (8)
	Other	4 (1)
**Self-rated health status**		
	Poor	16 (4)
	Fair	12 (3)
	Good	52 (13)
	Very good	20 (5)
	Excellent	0 (0)
**Years with type 2 diabetes**		
	<1 year	0 (0)
	1-2 years	8 (2)
	3-5 years	28 (7)
	6-10 years	20 (5)
	11-19 years	28 (7)
	≥20 years	4 (1)
	Declined to answer	12 (3)
**Facebook use**		
	More than once a day	56 (14)
	Once a day	32 (8)
	A few times a week	8 (2)
	Once a week	0 (0)
	Less than once a week	0 (0)
	Decline to answer	4 (1)

## Discussion

### Principal Results

The results of this study demonstrate the complexity of selecting a mechanism of health information communication on Facebook. Participants considered many factors before making a decision, including their own and the recipient’s characteristics and relationships, the content of the information, the communication purpose, and the attributes of a particular communication mechanism. These findings mirror findings of previous studies documenting patients’ general rationales for communicating health information [13-15], but add the dimension of the inherent features of specific communication mechanisms. It is thus imperative that consumer health IT designers create solutions responsive to these diverse rationales, better facilitating communication between patients and their social networks.

Our findings partially support previous literature focused on the use of Facebook groups for health information communication [23,35-40]. Similar to previous studies, participants in our study used Facebook groups for acquiring informational support (eg, explaining symptoms, treatments, medications) and emotional support (eg, providing encouragement, affirmations). Our qualitative interviews yielded discussion focused primarily on these rationales for Facebook group use. Moreover, as reported in a previous study, participants indicated using Facebook groups to express their opinions and experiences with health care centers, providers, and health insurance companies [39]. Other previously documented rationales for using Facebook groups for health information communication such as marketing health services and products [23,41], and supporting fundraising efforts for a health condition [22,37,41] were not explicitly mentioned in our qualitative interviews. This contrast in findings may have been because our interview probes focused on clinical and experiential forms of health information relevant to the individual.

In addition to supporting previous literature on the use of Facebook groups for health information communication, our study provided a novel, in-depth explication of how other communication mechanisms on Facebook are used for this purpose. As a whole, participants reported using the full range of available communication mechanisms, spanning those that are private versus public, synchronous versus asynchronous, and active versus passive to meet a range of communication needs. This general finding, that patients use a variety of mechanisms to communicate health information depending on the situation and intended recipient(s), parallels those of other studies of patients’ online and offline health information communication with social network members [17,50,51]. Despite these communication patterns, few existing consumer health IT solutions mimic and integrate this broad range of communication mechanisms. This is particularly true of consumer health IT solutions focusing on personal social networks such as Microsoft HealthVault [52] and CaringBridge [53], which are constrained by the types of communication mechanisms offered to users. Few support communication mechanisms beyond those that, in Facebook terms, equate with posting/commenting on a timeline and giving another individual full access to one’s account. There is a need to expand the communication mechanisms offered within consumer health IT to better encompass the range of mechanisms for which participants demonstrated utility in this study.

The use of multiple communication mechanisms on Facebook to communicate health information may arise from participants’ need to balance competing priorities. Understanding these balancing acts can provide insight into additional design opportunities for consumer health IT. Patients’ use of both active (eg, chat, posting and commenting on timeline) and passive (eg, tagging, sharing) mechanisms reflects their need to variously balance the desire to communicate health information against the effort required [16]. In this decision, the effort in question occurs at the time of the communication act. Thus, passive mechanisms, although not yet incorporated into existing consumer health IT solutions, may serve a unique purpose. Specifically, they would enable users to communicate emotional support (by liking), the relevance of specific information to an individual (by sharing or tagging), or their physical presence at a health-related event (by checking in) without a significant time investment. In contrast, while the communication-effort balance also manifests itself in the decision to use public versus private mechanisms, the effort in question occurs at the time of the response to the initial communication act. For example, participants in our study spoke about the decision not to use public communication mechanisms such as posting on a timeline because of the multitude of responses that would need to be addressed. To mitigate the effort required to sift through and make sense of responses, natural language processing capabilities could be embedded within consumer health IT, grouping and summarizing similar types of feedback. A user could then address a group of similar messages with one response. Participants also noted that the choice between public and private mechanisms requires balancing the desire to obtain the fruits of communicating (eg, information or social support) with the desire to manage self-presentation [30] or the presentation of others. Design opportunities, including providing proactive feedback to dissuade social network members from providing negative responses [30], offering transparent granularity in sharing controls [54], and enabling anonymous posting within one’s social network [55], have been offered as means of alleviating the necessity for such balance. These could be augmented with ways of illustrating who in a recipient’s social network would have access to posted information so that the communicator can work to preserve not only their own, but also their social network member’s self-image.

Beyond the design recommendations discussed above, there are likely opportunities to integrate or extend existing functionality within Facebook to better meet patient needs for consumer health IT supporting health information communication. For example, participants in our study indicated that their reaction to social network members’ feedback is dependent on mood. Facebook currently has the capability of allowing users to indicate their mood and to link this mood to a specific post. This type of functionality could be integrated into consumer health IT to allow people to provide their current emotion as related to a piece of information, better enabling them to receive appropriate and supportive feedback. Similarly, our participants stated that their use of certain Facebook mechanisms is motivated by a need to ensure that the social network member will receive the message. Facebook currently informs users if a message was received through the chat or private messaging function (by displaying the word “read”) and through groups (by displaying who has seen the post). This functionality could also be integrated into consumer health IT, along with an additional layer that enables users to visualize the reliability of a social network member receiving a message through a given communication mechanism. As a third example, participants expressed the importance of communicating information that was relevant to themselves and to their social network members. At present, Facebook allows users to control input to their newsfeeds by individual (whose posts are prioritized). To meet the needs of our participants, consumer health IT should enable users to prioritize information received by both user and topic. Moreover, to help users not only receive but also send relevant information, text mining capabilities could be extended to offer suggestions on which social network members should be tagged in a post.

### Limitations

This study is subject to several limitations. First, the retrospective interview approach to data collection is subject to respondent recall bias. We attempted to mitigate this bias in the qualitative portion of our study by asking participants to review their Facebook communications prior to engaging in the interview. Second, the study focused only on individuals with one diagnosis (ie, type 2 diabetes), although some reported co-morbidities. It is likely, however, that the results have some generalizability to individuals with other chronic health conditions requiring similar levels of daily engagement. Third, participants in this study all lived in or were residents of the United States. It is possible that individuals living in other countries would approach health information communication on Facebook differently. However, our sample contained cultural diversity in the sense that it purposefully oversampled individuals identifying as racial and ethnic minorities. Finally, it is important to note that understanding communication mechanism use on Facebook is only one of multiple ways of understanding patient needs for consumer health IT focused on supporting health information communication between patients and their social networks. The lessons learned from this study should be combined with other needs assessments within a participatory design process when developing a specific consumer health IT solution.

### Conclusions

When choosing a mechanism for health information communication, participants consider multiple factors that intersect in complex ways. Factors included what information they intended to share, what they were trying to accomplish, attributes of technology, and attributes and communication practices of their social network. There is a need for consumer health IT communication mechanisms to allow for a range of choices to suit the intersectionality of participants’ rationales. Technology that better meets patients’ needs will lead to better self-management of health conditions, and therefore, improve overall health outcomes. This study demonstrates that this intersectionality leads to a range of preferred communication mechanisms beyond those commonly available within existing consumer health IT solutions. As design of these systems evolve, there is a need to meet demand for a bundle of mechanisms facilitating communication between an individual and multiple types of social networks—communication mechanisms that vary in terms of level of effort, degree of synchronous communication, and privacy control. Future research could include designing consumer health IT solutions that have these specific communication mechanisms and then testing the solution with patients of varying health conditions. Additionally, by describing participants’ rationales, we can infer the underlying dynamics of participants’ choice of communication method. However, this interpretation is at an aggregate level across participants. Data from our interviews do not tell us systematically who is making what type of decisions under what circumstances. Another promising direction for future research will be to build a model of health information communication choices. Analysis of the results from the larger study from which data for this study were drawn will contribute to this research.

## Appendix

Multimedia Appendix 1

## Abbreviations

IT: information technology
SNS: social networking site
