# Supplementary material for: Mechanisms of Communicating Health Information Through Facebook: Implications for Consumer Health Information Technology Design
Source: J Med Internet Res. 2016 Aug 11;18(8):e218. doi: 10.2196/jmir.5949 (PMC4999533; doi:10.2196/jmir.5949)
Supplement: Multimedia Appendix 1 [file jmir_v18i8e218_app1.pdf]

Appendix

Table A: Communication mechanisms on Facebook

**Definitions and details regarding communication mechanisms**		
Types of communication mechanisms		Active: generally used to generate customized and relatively detailed content
		Asynchronous: enable communication to take place over longer stretches of time
		Passive: generally used to draw attention to preexisting content
		Private: generally targeted to one social network member
		Public: generally targeted to multiple social network members
		Synchronous: enable real time exchange of information
Posting vs. commenting		Posting and commenting are two actions relevant to multiple Facebook communication mechanisms. Posts enable a user to initiate a conversation, whereas comments enable a user to respond to an ongoing conversation.
Privacy settings		By changing Facebook's default privacy settings, users can tailor the degree to which communication via public modes is restricted to specific social network members. The privacy of group-based communication may similarly be restricted through privacy settings set by the group administrator
**List of Facebook communication mechanisms**		
Type of communication mechanism	Facebook communication mechanism	Description of communication mechanism
• Active<br>• Private<br>• Synchronous	Messaging/Chat	Messaging and chatting on Facebook are two parts of the same communication mechanism. The communication mechanism functions as messaging when the intended recipient is offline and as chatting when the intended recipient is online. Messaging is similar to emailing. The intent of a message is to have an asynchronous conversation with one person or a group. Chatting on Facebook is similar to instant messaging. Chat facilitates asynchronous conversation between two logged-on individuals. Similar to messages, chats can be sent to one person or a group.
• Active<br>• Asynchronous<br>• Public	Post or comment to closed group	Closed groups are spaces that can be joined by invitation or through moderator approval. Anyone can find the group and see its members, but only members can see content.

	Post or comment to secret group	Secret groups can only be joined through an existing member’s invitation. Current and former members can find the group, but only current members can see its members and content.
	Post or comment on own timeline	A timeline is an individual user’s personal space on Facebook and has some similarities to a profile or a blog. Depending on the Facebook user’s privacy settings, the content on their timeline may be visible only to them, a subset of their friends, all their friends, or all Facebook users.
	Post or comment on friends’ timelines	A Facebook user “friends” an individual with whom the Facebook user has established a reciprocal relationship. A friend may give the Facebook user permission to add content to the friend’s timeline.
	Posts or comment to pages	A page is a personal space for a business or organization as opposed to an individual. Facebook users can opt to receive content from pages or may add content if allowed by the moderator.
• Passive<br>• Public<br>• Synchronous	Share	The “share” button on Facebook allows users to repost content other users have posted to Facebook or repost content to Facebook found on external websites integrated with Facebook (i.e., websites displaying the “share” option on their page). Users can “share” posts to their timeline, friends’ timelines, groups, or pages.
	Like	The “like” button on Facebook allows users to provide positive feedback on other users’ posts or comments.
	Check-in	The “check-in” button on Facebook allows users to post their current physical location to their timeline using the GPS capability on their devices.
	Tag	The “tag” feature on Facebook allows users to communicate the relevance of specific personal content (e.g., pictures, posts, comments) to specific Facebook friends.
